# Ischemic Heart Disease and Liver Cirrhosis: Adding Insult to Injury

**DOI:** 10.3390/life12071036

**Published:** 2022-07-12

**Authors:** Irina Gîrleanu, Anca Trifan, Laura Huiban, Cristina Muzîca, Oana Cristina Petrea, Ana Maria Sîngeap, Camelia Cojocariu, Stefan Chiriac, Tudor Cuciureanu, Irina Iuliana Costache, Carol Stanciu

**Affiliations:** 1 Depatment of Internal Medicine, Grigore T. Popa University of Medicine and Pharmacy, 700115 Iaşi, Romania; irina.girleanu@umfiasi.ro (I.G.); huiban.laura@yahoo.com (L.H.); lungu.christina@yahoo.com (C.M.); oana.stoica@umfiasi.ro (O.C.P.); anamaria.singeap@umfiasi.ro (A.M.S.); camelia.cojocariu@umfiasi.ro (C.C.); stefan.chiriac@umfiasi.ro (S.C.); tudor.cuciureanu@umfiasi.ro (T.C.); ii.costache@yahoo.com (I.I.C.); stanciucarol@yahoo.com (C.S.); 2 Institute of Gastroenterology and Hepatology, Saint Spiridon University Hospital, 700115 Iaşi, Romania; 3 Cardiology Department, Saint Spiridon University Hospital, 700115 Iaşi, Romania

**Keywords:** coronary artery disease, liver cirrhosis, prevalence, liver transplantation, treatment

## Abstract

The link between heart and liver cirrhosis was recognized decades ago, although much data regarding atherosclerosis and ischemic heart disease are still missing. Ischemic heart disease or coronary artery disease (CAD) and liver cirrhosis could be associated with characteristic epidemiological and pathophysiological features. This connection determines increased rates of morbidity and all-cause mortality in patients with liver cirrhosis. In the era of a metabolic syndrome and non-alcoholic fatty liver disease pandemic, primary prevention and early diagnosis of coronary artery disease could improve the prognosis of liver cirrhosis patients. This review outlines a summary of the literature regarding prevalence, risk assessment and medical and interventional treatment options in this particular population. A collaborative heart–liver team-based approach is imperative for critical management decisions for patients with CAD and liver cirrhosis.

## 1. Introduction

The association between liver cirrhosis (LC) and coronary artery disease (CAD) is still a matter of debate. Common pathogenic mechanisms (vascular inflammation, endothelial dysfunction, a procoagulant status) were recognized to be associated with developing atherosclerotic lesions in patients with LC [1,2].

Cardiovascular abnormalities in LC are associated with a hyperdynamic hemodynamic status, increased resting cardiac output and a blunt ventricular response to stress, systolic and diastolic dysfunction, along with electrophysiological abnormalities such as QT interval prolongation [3].

**Figure 1:**
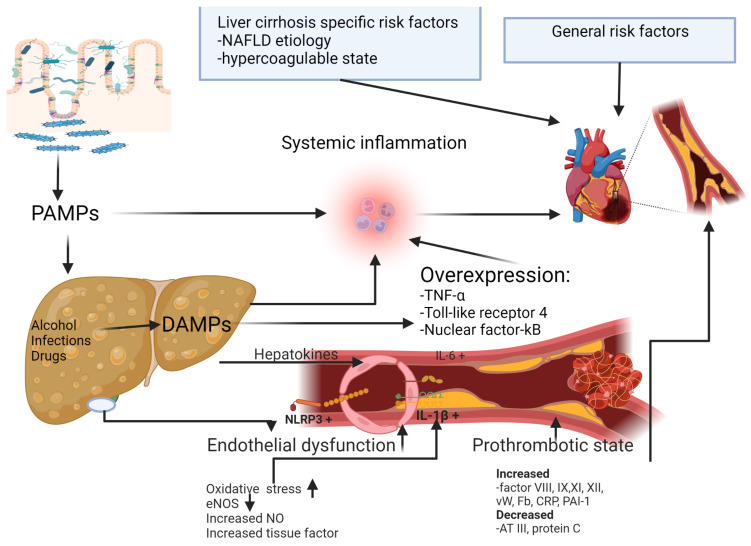
Pathogenic mechanism of coronary artery disease in patients with liver cirrhosis. Abbreviations: ATIII: antithrombin III; CRP: C-reactive protein; DAMPs: damaged-associated molecular patterns; Fb: fibrinogen; IL-6: Interleukin 6; IL-1β: Interleukin 1β; NAFLD: non-alcoholic fatty liver disease; NLRP3: pyrin domain-containing protein 3; NO: nitric oxide; PAI-1: plasminogen activator inhibitor-1; PAMPs: pathogen-associated molecular patterns; TNF-α: tumoral necrosis factor; vW: von Willebrand factor.

LC is characterized by increased oxidative stress and low nitric oxide (NO) bioavailability, resulting in vascular inflammation, endothelial dysfunction, increased production of tissue factor, hypercoagulability and vascular smooth-muscle proliferation [4,5]. To all these factors, hepatic inflammation is superimposed by overexpression of TNF-α, angiotensin II, toll-like receptor 4 and nuclear factor-kB [1]. Portal hypertension is associated with increased gut permeability and pathogen-associated molecular patterns (PAMPs) that activate an immune response and increase systemic inflammation [6]. Alcohol consumption, infections or drug-induced liver injury cause hepatocyte apoptosis with secondary release of damage-associated molecular patterns (DAMPs) with an important role in increasing the inflammatory response [7] (Figure 1).

Moreover, in cirrhotic patients, increased peripheral NO induces splanchnic vasodilatation. Pulse wave velocity measurement is a non-invasive tool for early atherosclerosis diagnosis in direct relation with intima–media thickness in patients with LC despite splanchnic vasodilatation, confirming the fact that these patients are not protected from developing atherosclerotic lesions [8].

Moreover, the effects of diabetes mellitus (DM), smoking, hypertension and metabolic syndrome, in different combinations, accelerate the process of atherosclerosis and can lead to acute coronary syndrome [3,4,5]. Additionally, current evidence suggests that when the traditional risk factors for CAD are controlled, cirrhotic patients remain at high risk of developing CAD [8].

Data published until now have demonstrated that the development of CAD in patients with LC is associated with poor prognosis and high mortality rates, including patients on the liver transplant waiting list [9]. Even if important progress has been made regarding early diagnosis and treatment of CAD in recent years, for the cirrhotic population there are still many unresolved issues. Therefore, we aimed to present an updated comprehensive narrative review on the currently available literature related to CAD and LC. 

## 2. Epidemiology, Clinical Presentation and Risk Factors

### 2.1. Epidemiology of CAD in LC

A few decades ago, patients with liver cirrhosis were considered protected from developing ischemic cardiac disease due to low blood pressure and low cholesterol levels [9]. However, recent data confirmed that CAD are more prevalent in the cirrhotic population compared with the general population [9,10]. The prevalence of CAD in patients with liver cirrhosis ranges between 1.7 and 27%, and it is associated with high mortality during and after liver transplantation surgery [11,12,13,14,15]. This wide prevalence variation could be explained by the heterogeneity of the cirrhotic population and the different diagnostic methods and CAD definitions. Autopsy studies have demonstrated that patients with LC have less advanced atherosclerotic plaques and a low prevalence of calcified atherosclerotic lesions versus non-cirrhotic patients. However, these data were not confirmed by imagistic studies, which confirmed that atherosclerotic lesions are at least as frequent in patients with LC as in general population, especially in the decompensated stage of the disease. The majority of studies evaluating the CAD epidemiology in LC differed in their control groups, and they involved different patients in terms of cardiac symptoms and history, all these representing an important statistical bias. The knowledge of the prevalence of CAD, particularly of anatomically confirmed CAD, in patients with LC is very limited.

Some studies demonstrated a low prevalence (1.7%) of CAD in patients with LC, despite the presence of atherosclerotic risk factors [16,17]. It has to be mentioned that these were retrospective studies, the prevalence of CAD was a secondary endpoint, and most of the patients were diagnosed with viral decompensated LC. When the diagnosis was based on computed tomography (CT) angiography, the prevalence of asymptomatic obstructive CAD was 7.9% in a cohort including the majority of the patients with decompensated viral B liver cirrhosis [10], as was demonstrated in more than 1000 Korean patients.

A recent meta-analysis including almost 13,000 patients concluded that the prevalence of obstructive CAD among patient with LC reached 12.6%, similar to the general population [11], and the highest prevalence was reported in cirrhotic patients aged over 50 years old. Moreover, non-obstructive CAD has a higher rate in patients with LC [10] compared with the general population (30.6% vs. 23.4%, *p* = 0.001). Additionally, acute coronary syndrome had a prevalence of 2.81 per 1000 person-years and was higher in patients with ascites. 

### 2.2. Clinical Presentation of CAD in LC

Liver cirrhosis, especially in the decompensated stage, modifies the clinical presentation and the main symptoms of CAD. Asymptomatic presentation is very common in a cirrhotic population; only a minority of patients with LC who presented with acute myocardial infarction declared chest pain, compared with 72% of patients from the general population [10], although these patients were more likely to report dyspnea, a frequent symptom in patients with LC and grade 2 or 3 ascites or pleural effusion. Thus, recognition of ischemia in patients with LC requires a high index of suspicion, considering that CAD presents atypically in this special population. Chronic coronary syndrome is more difficult to diagnose in patients with LC as most of them have no angina symptoms. They can present with dyspnea on exertion or effort thoracic anterior pain. Differential diagnosis should be made with porto-pulmonary hypertension, pulmonary embolism, pleural effusion, or pneumonia. Special attention should be paid in cirrhotic patients admitted with hemorrhagic complications, as anemia could precipitate an acute coronary syndrome and routine administration of vasoactive treatment—terlipressin—could aggravate the cardiac ischemia.

### 2.3. Risk Factors for CAD in LC

Risk assessments are based on population studies and are scant for cirrhotic populations. The major cardiovascular risk factors for CAD development are: arterial hypertension, diabetes mellitus, hyperlipidemia, obesity and smoking. DM, obesity and smoking were recognized as risk factors for CAD in patients with LC, along with age greater than 50 years old [9,13,18]. Diabetes mellitus is one of the main cardiovascular risk factors identified in patients with LC, the majority of the patients having asymptomatic CAD, although it is not clearly established if DM is more atherogenic in patients with LC [19]. It was demonstrated that LC is associated with low LDL levels, elevated HDL-2 cholesterol concentration and decreased adipokine levels, all of these leading to improvement in the lipid profile [14].

Controversies still remain regarding the role of heavy alcohol consumption on CAD prevalence. Patel et al. [12] demonstrated that patients diagnosed with alcoholic LC have a lower incidence of severe CAD. In contrast to these findings, Gologorsky et al. [18] found no evidence for low prevalence of CAD in patients with alcoholic LC in a large cohort including almost 6000 patients.

Patients with LC exemplify the shortcomings of risk assessments from population data, as they have low values of arterial pressure and low cholesterol levels. However, these risk prediction scores can be improved by adding liver-specific variables, such as model for end-stage liver disease (MELD) score, albumin level or liver fibrosis scores. Additional risk markers may help to refine atherosclerotic cardiovascular risk (Table 1). Coronary artery calcification can be used to identify CAD in patients with LC, especially in those evaluated for LT. Similarly, the prognostic significance of various circulating biomarkers, such as C-reactive protein, cardiac troponine and natriuretic peptides, may be lower than that of the general population.

Such novel serum biomarkers as matrix metalloprotease 9 (MMP-9), pentraxin 3, growth differentiation factor 15 (GDF-15), myeloperoxidase (MPO) and monocyte chemoattractant protein 1 (MCP-1) were proved to increase CAD risk prediction in a general population. MMP-9, GDF-15 and pentraxin 3 are involved in liver angiogenesis and fibrogenesis processes [20,21,22,23]. MCP-1 is an inflammatory chemokine associated with LC complications and mortality [24]. None of these novel biomarkers were evaluated for cardiovascular risk prediction in patients with LC. It has to be mentioned that finding such a biomarker in cirrhotic patients will be very difficult as most of them reflect fibrosis or inflammation, processes highly active in liver cirrhosis. However, it remains to be determined whether incorporation of these biomarkers into clinical care will affect the outcomes of cirrhotic patients. Entirely new cardiovascular risk models may be needed in LC, as the Framingham risk equation underestimates risk in liver transplant recipients, and modified equations have not been validated in this population. 

Recently, new data were published regarding the role of non-invasive liver fibrosis scores and the risk of CAD. These studies suggest that advanced liver fibrosis is associated with a high risk of CAD [25], and moreover with complexity of CAD [26].

## 3. Etiology of Liver Cirrhosis and CAD

There is a well-known association between *non-alcoholic fatty liver disease* (NAFLD) and an increased risk of ischemic heart disease up to 21.6% compared with 5.0% in patients with LC from other etiologies [27]. It was demonstrated that patients with NAFLD have more complex CAD with more than three vessels involved [13], due to poor coronary collateral development [28,29] and vulnerable plaque formation [30,31]. Even if NAFLD has common risk factors with metabolic syndrome and the prevalence of CAD is higher in patients with NAFLD, data regarding the frequency of CAD in patients with NAFLD and advanced fibrosis, including LC, are scant. In a large cohort including patients diagnosed with CAD by coronary angiography and evaluated for NAFLD and fibrosis using transient elastography and controlled attenuation parameters, obstructive CAD was less frequent in patients with advanced liver fibrosis compared with mild fibrosis [32]. Recent studies demonstrated that advanced fibrosis secondary to NAFLD diagnosed by noninvasive serum markers is associated with CAD [2]. On the contrary, in an Asian population, NAFLD liver cirrhosis was associated with a reduced prevalence of CAD [33], confirming that there are still some puzzle pieces missing in the larger picture of NAFLD and CAD. It has not yet been demonstrated that NAFLD is an independent risk factor for CAD in patients with LC; therefore, there are no clear recommendations for screening for CAD in all patients with NAFLD liver cirrhosis. 

Data regarding the influence of *chronic alcohol consumption* on the risk of CAD are contradictory. Patel et al. demonstrated in 420 cirrhotic patients that those with alcoholic cirrhosis had a lower incidence of CAD compared with other etiologies of LC [12]. It has to be mentioned that the patients diagnosed with NAFLD liver cirrhosis were not excluded from the control group. Considering that NAFLD is an independent risk factor for CAD in patients with or without metabolic syndrome, the results of this study should be carefully interpreted [34]. 

The association between *viral hepatitis* and cardiovascular risk is also controversial. Previous studies reported no relationship between viral hepatitis and carotid arteries’ atherosclerotic lesions; moreover, viral C etiology was negatively correlated with CAD [35]. On the contrary, recent data have confirmed that virus C infection is associated with increased systemic inflammation, insulin resistance, dyslipidemia, carotidian atherosclerotic plaques and hepatic steatosis [36,37,38]. Additionally, chronic hepatitis C was associated with an increased epicardial fat thickness and carotid intima–media thickness, and these were in direct positive correlation with LC severity assessed by Child–Pugh class and CAD [39]. Moreover, the antiviral treatment with virological response was associated with a significant decrease in atherosclerotic plaques [40,41]. 

*Primary biliary cholangitis* (PBC) is characterized by an increased level of cholesterol and chronic systemic inflammation, although the influence of this dyslipidemia in the development of CAD was not clearly demonstrated. In a retrospective cohort study, Wang et al. demonstrated that only arterial hypertension is an independent risk factor for CAD in patients with PBC [42]. Three large cohorts, including more than 500 cirrhotic patients, also found no relationship between PBC and the CAD development [43,44,45]. On the contrary, a meta-analysis of only four observational studies demonstrated that PBC is associated with a 57% excess risk of CAD, and patients with PBC should receive appropriate treatment for traditional cardiovascular risk factors [46].

## 4. CAD Diagnosis in Liver Cirrhosis

Considering the difficulties in the diagnosis of CAD in patients with LC, testing is recommended in symptomatic patients and in those asymptomatic needing moderate to high-risk surgery, including LT. Identifying the best method for CAD screening in patients with LC is still a matter of debate.

For the diagnosis of acute coronary syndrome, the current guidelines should be followed [47], and myocardial infarction should be diagnosed according to the fourth Universal definition of Myocardial Infarction [48]. In patients with chest pain, high-sensitivity troponin level is a reliable marker for myocardial ischemia [49]. In patients with LC, troponin levels may be elevated secondary to myocardial damage unrelated to obstructive CAD [50] as a marker of cirrhotic cardiomyopathy [51] in direct relation to liver disease severity [52]. 

Coronary angiography is the gold standard for CAD diagnosis, although, in patients with LC, it is an invasive procedure with a high risk of complications: bleeding, acute kidney injury, etc. Considering these particularities, non-invasive tests are preferred for CAD diagnosis in symptomatic cirrhotic patients or those who are asymptomatic undergoing moderate or high-risk surgery. In the setting of acute coronary syndrome, coronary angiography is the main method of diagnosis including in patients with LC. 

Cirrhotic patients with acute coronary syndrome, symptomatic angina refractory to treatment, and those asymptomatic with a non-invasive positive test have indication for *invasive coronary angiography* [53]. Transradial access for cardiac catheterization in patients with LC is safer compared with femoral access [54].

The European Association for the Study of the Liver recommends electrocardiogram and echocardiography in all LT candidates, and in those older than 50 years, with multiple cardiovascular risk factors an exercise stress test is indicated [55], although, the American Association for the Study of Liver Disease indicates that all LT candidates should benefit from a stress echocardiography regardless of the cardiovascular risk factors [56]. 

The non-invasive methods for CAD diagnosis are represented by stress echocardiography, computer tomography angiography (CTA) with coronary artery calcium (CAC) scoring, cardiac magnetic resonance imaging (MRI), and single-photon emission computed tomography myocardial perfusion imaging [19]. 

*Exercise testing* is difficult to perform in patients with LC as it is frequently limited by the inability of these patients to reach diagnostic workloads. Second, exercise testing in LC is often limited by baseline electrocardiographic abnormalities and non-selective beta-blocker treatment. Third, most existing data were derived from studies of transplant candidates—the extent to which these data can be generalize to all cirrhotic patients is uncertain. It has to be mentioned that in patients with LC, ST segment depression can be identified in exercise tests in the absence of significant CAD, as a consequence of a disorder in the myocardial microcirculation secondary to vasodilatation mediated by the coronary artery endothelium [57]. Even if exercise stress testing is possible in a patient with LC, its sensitivity is poor. 

Other stress tests with other drugs (such as terlipressin and metariminol) or volume challenge still remain in the research field. *Dobutamine stress echocardiography* (DSE) has low specificity as it is less likely for the patients to achieve target heart rate during pharmacological-induced stress [58]. In a study including more than 600 cirrhotic patients, DSE had a sensitivity of only 19% and a specificity of 90% (negative predictive value 84%, positive predictive value 29%) [59]. This low sensitivity is explained by chronotropic incompetence in patients with LC. 

*Coronary artery calcium score* and *computed tomography angiography* (CTA) may offer significant advantages over functional imaging modalities in the setting of liver cirrhosis. In a cohort of 147 cirrhotic patients on the liver transplant waiting list, more than half of the patients were classified in the moderate/high-risk group [17]. Compared with other methods of CAD evaluation in asymptomatic patients with LC, CAC is the most useful predictor and can be used in order to triage the patients for angiography [60]. This method has a 47% positive predictive value and 99% negative predictive value for diagnosis of significant coronary stenosis [19]. CAC measures the total amount of coronary calcium, expressed as Agatston units. Values greater than 400 are associated with severe CAD [61], and could identify the high-risk cirrhotic patients that could benefit from coronary angiography. 

In patients with suspected CAD, *stress cardiac MRI* could be used for assessing the myocardial scars and to identify the viable myocardial tissue for revascularization procedures (62). Stress cardiac MRI has a sensitivity of 77% and a specificity of 88% for detecting significant coronary stenosis (>50%) [62], although data for patients with LC are scant, and due to its high cost the utility in routine evaluation of patients with LC is limited. 

**Figure 2:**
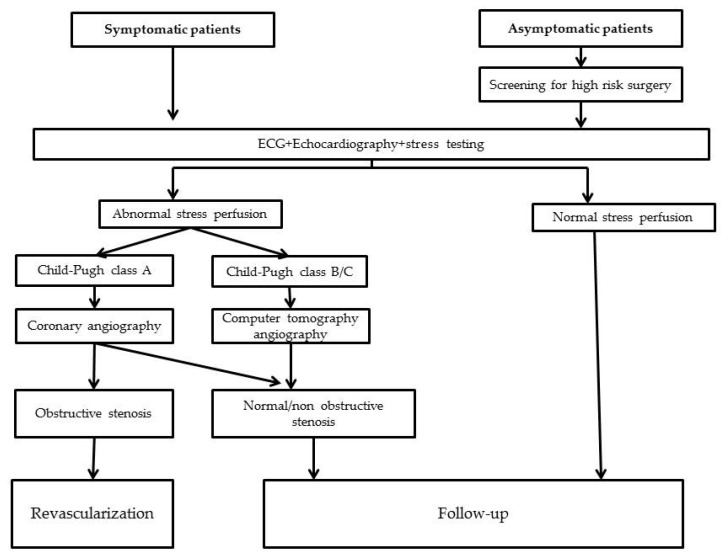
Diagnostic algorithm for patients with LC and CAD.

The diagnosis of CAD in patients with LC could be challenging due to overlapping symptoms, especially in decompensated LC. Chest pain could be secondary to pleuritis, pneumonia or musculoskeletal disorders. Dyspnea has many causes in cirrhotic patients: pleural effusion, porto-pulmonary hypertension, hepato-pulmonary syndrome, pneumonia, pulmonary embolism, large ascites, or anemia. Moreover, in decompensated LC patients, low-voltage ECG is frequent, and transthoracic echocardiographic examination is limited by pleural and pericardial effusion and ascending diaphragms. All these elements concur to a delayed diagnosis of CAD in patients with LC. A diagnostic algorithm is presented in Figure 2.

## 5. Management of CAD in Liver Cirrhosis

### 5.1. Medical Therapy

The management of CAD in patients with LC is an unsolved issue. Although medical therapy is the cornerstone of CAD treatment, challenges exist in cirrhotic patients, considering the fact that patients with LC are under-represented in clinical trials and, as such, the evidence to support recommendations is limited. 

Such *nonselective beta-blockers* as propranolol and carvedilol are frequently used in patients with LC for variceal bleeding prophylaxis due to their ability to decrease the portal vein pressure. In patients with CAD, the benefit of beta-blocker treatment is well established. It reduces myocardial ischemia and is effective in improving symptoms. Peuter et al. demonstrated that nonselective beta-blockers are associated with a higher decrease in mortality and vascular events than cardioselective receptor blockade in patients with CAD [63], although data evaluating the role of beta-blockers on CAD treatment in patients with LC are lacking. 

*Antiplatelet therapy* could be indicated in patients with LC. Even if this therapy is associated with an increased risk of non-portal hypertension-related bleeding, there is no influence on mortality [64]. Moreover, most of the cirrhotic patients have thrombocytopenia, although platelet aggregation is normal, as was demonstrated by aggregometry methods [65]. Caution should be taken in patients with platelets less than 50 × 10^9^/L, as those are prone to develop bleeding complications [66].

*P2Y_12_ inhibitors* block adenosine-diphosphate-induced platelet aggregation. Clopidogrel is an irreversible inhibitor of P2Y_12_ and the liver is the site of metabolic activation. In patients with compensated LC, Child–Pugh class A, clopidogrel pharmacokinetics is not influenced [67]. No dose adjustments should be made in these patients. However, in patients with decompensated LC, Child–Pugh class C, clopidogrel treatment inhibited platelet function with no increase in the side effect rate [65]. Prasugrel, another P2Y_12_ inhibitor, could represent a safe treatment in patients with LC, as it has no hepatic metabolism. Ticagrelor, a reversible P2Y_12_ inhibitor, has hepatic metabolism, being activated by the liver cytochrome 3A4 enzyme, and it is not recommended in patients with Child–Pugh class C liver cirrhosis [68]. Even if Ticagrelor is a reversible P2Y_12_ inhibitor, the effect on platelet inhibition is prolonged with increasing LC severity [69]. Considering all these data, in cirrhotic patients presenting with acute coronary syndrome undergoing PCI, prasugrel is the preferred drug, as it has benefits over ticagrelor in lowering mortality and stroke rate without increasing the bleeding risk [70]. There are only case reports regarding the use of glycoprotein IIb/IIIa inhibitors in patients with LC. Eptifibatide was associated with severe thrombocytopenia in patients with LC and an increased hemorrhagic risk [71].

*Dual antiplatelet therapy* is recommended for at least 12 months after drug-eluting stent insertion and for at least 1 month after a bare metal stent placement [53]. There are few studies evaluating the safety of this drug association in patients with LC. In a retrospective cohort comparing cirrhotic patients with or without aspirin and clopidogrel treatment, it was demonstrated that the rate of fatal variceal bleedings was very high (12.5% vs. 6.3%), concluding that this treatment should be restricted to cirrhotic patients without esophageal varices [72]. In studies performed on national databases, it was demonstrated that patients with LC have a higher risk of bleeding complications on dual antiplatelet therapy and an individualized approach is needed [73,74]. 

Controversy surrounds the use of *statins* in patients with LC. There are no studies confirming the benefits of statins in reducing major vascular events in patients with LC, although statins can be safely used in patients with LC. Recent studies confirmed that simvastatin decreases portal pressure [75]. Statins decrease hepatic fibrogenesis, protect from severe LC complications and even reduce the risk of hepatocellular carcinoma by improving inflammation and endothelial dysfunction [76]. It has to be mentioned that data on the use of statins in patients with decompensated LC are very limited. 

Considering all these data, Patel et al. [77] demonstrated that aspirin and statins are safe in patients with decompensated LC, and they can be indicated in patients that associate CAD when the benefits of cardiovascular events prevention outweigh the risk of bleeding. 

*Angiotensin-converting-enzyme inhibitors, angiotensin receptor blockers and direct renin inhibitors* are not indicated in patients with LC, especially in those in the decompensated stage of the disease due to the effect of vasodilation, which could increase the risk of acute kidney injury, including hepato-renal syndrome, and predispose the patients to electrolyte disturbances. The risk of hyperkaliemia in cirrhotic patients treated with angiotensin-converting-enzyme inhibitors is 5.2-fold greater compared with that in patients without LC [78]. Patients with LC have an activation of the renin angiotensin aldosterone system and of the sympathetic nervous system, causing renal vasoconstriction, impaired renal perfusion and increased sodium retention. ACE inhibitors or sartans can further impair glomerular filtration due to reduced filtration pressure, and they should be used very cautiously in cirrhotic patients [79,80].

There is little information regarding the safety of other specific anti-angina drugs in patients with LC. *Ranolazine* inhibits the late inward sodium current and has as a side effect QTc prolongation. *Ivabradine* combined with carvedilol could improve diastolic dysfunction and survival rate in patients with liver cirrhosis [81], also decreasing the complication rate. 

### 5.2. Revascularization in Patients with CAD and Liver Cirrhosis

The choice of medical therapy alone or revascularization with percutaneous coronary intervention (PCI) or coronary artery bypass (CABG) in symptomatic patients with LC is controversial. In the absence of dedicated clinical trials, cirrhotic patients presenting with acute coronary syndrome undergo the same invasive approach as those with normal liver function. 

#### 5.2.1. Percutaneous Coronary Intervention (PCI)

Mortality in patients with CAD and LC is higher than in the general population, although the mortality rates in this population increased during the last decade [82]. PCI in patients with LC is associated with a higher procedural risk, more frequent cardiogenic shock, cardiac arrest and gastrointestinal bleeding, along with a fivefold increase in acute kidney injury compared with general population [83].

An observational study evaluating the trends in PCI evolution between 2003 and 2016 in the United States demonstrated that PCI procedures increased among patients with LC and decreased in the general population [84]. These data could be explained by a better recognition of cardiovascular risk factors associated with early primary prevention methods in the general population and the lack of a precise cardiovascular score in a cirrhotic population. In this large study, the rates of vascular complications were similar in patients undergoing PCI with or without LC. 

A large retrospective cohort including cirrhotic patients with LC undergoing PCI demonstrated that this procedure is associated with high bleeding complications and mortality, especially when bare-metal stents were used [83] compared with the general population. It has to be mentioned that 77% of the procedures were performed on an emergent basis and 15% for ST-elevation myocardial infarction presentation, although no data on the severity of LC were reported. Bare-metal stents were preferred in patients with LC due to short-term use of dual antiplatelet drugs, even if the data published did not demonstrate that bare-metal stents are associated with a lower bleeding risk compared with drug-eluting stents in patients with LC [83]. The long-term safety of PCI in patients with LC was evaluated in a cohort of 64 patients, with the majority in the decompensated stage of the disease. This study demonstrated that cirrhotic patients with CAD diagnosed before liver transplantation have an increased mortality after liver transplantation, as these patients have multivessel CAD. One of the largest retrospective studies including patients with LC and CAD demonstrated that the mortality rate was not influenced by PCI, suggesting that the revascularization in this special population is not associated with a major benefit in terms of survival, given the high rate of liver-related mortality [85]. Long-term data confirmed that the rate of adverse events is higher in cirrhotic patients undergoing PCI for CAD, with a severe bleeding rate of 23% and a rate of acute kidney rate of 26%, of whom patients needed renal replacement, compared with the group that received medical treatment. After 1 year of follow-up, the bleeding rate was doubled in patients receiving PCI [86]. The highest rate of complication was demonstrated in patients with decompensated LC, especially ascites and hepatic encephalopathy, before PCI [86]. Considering all these data, the benefit of this procedure, in asymptomatic decompensated cirrhotic patients is still under debate. 

There had been a preferential approach of opting for bare-metal stents in patients with LC because of earlier endothelialization and shorter dual antiplatelet therapy duration times. However, several clinical trials have demonstrated the superior outcomes of second-generation drug-eluting stents compared with bare-metal stents [84,85,86,87,88]. Coronary artery stenting and dual antiplatelet therapy are associated with an independent risk of variceal bleeding [72]. A history of variceal bleeding or presence of varies confers an increased risk, and primary or secondary prophylaxis of variceal bleeding is recommended prior to cardiac catheterization. 

#### 5.2.2. Coronary Artery Bypass Grafting (CABG)

Cardiac surgery is associated with a high risk of mortality in patients with LC, with an overall mortality rate of 26% [89]. Two large patients cohorts, the first one including patients evaluated between 1998 and 2004, and the second including those admitted between 2002 and 2014, demonstrated that coronary artery bypass grafting procedures in patients with LC increased, although they were associated with high morbidity and mortality risk [90,91], even if there is a tendency of decreasing in recent years. Bleeding complications were the most frequent cause of morbidity after CABG, and the patients in a decompensated stage of LC were more prone to developing complications and had increased in-hospital mortality. The highest mortality risk was identified in patients with a MELD score > 13 and Child–Pugh class more than 7 points [92], and these patients could benefit more from other methods for revascularization.

The waiting period from CABG and liver transplantation is still a matter of debate. This period varies between 12 h and 18 days. The data published until now conclude that we have to wait more than 10 days until liver transplantation after CABG [92].

Considering all these data, LC should be included in the cardiac risk assessment, and the decisions regarding the best option should include a combination between liver disease severity models and cardiovascular risk scores. 

## 6. Conclusions

Patients with LC and CAD constitute a high-risk population with unique epidemiological and pathophysiological characteristics. Considering the increasing prevalence of alcoholism, diabetes mellitus, obesity and non-alcoholic liver disease, the prevalence of CAD in LC is on the rise. The prognostic factors for CAD in LC have to be clarified, along with the role of screening programs and the most appropriate interventional preventive regimes. Data on the safety and efficacy of antiplatelet drugs in LC patients are derived from observational studies in patients with mild to moderate LC, and we have limited data on patients with severe decompensated LC. Additionally, the optimal duration of dual antiplatelet therapy in LC patients still remains to be defined, along with the limits of interventional revascularization procedures.

The data we have until now confirm the utility of non-invasive methods of diagnosis, and the safety and efficacy of beta-blockers and statins in patients with LC and CAD. Treatment decisions should balance between bleeding and thrombotic risks, especially in Child–Pugh C cirrhotic patients.

A heart–liver team-based approach is imperative for critical management decisions for this patient population, and clinicians need to become more aware of the association between CAD and LC.

## Figures and Tables

**Table 1:** Risk factors for cardiovascular events.

General Population Risk Factors
Age > 50 years
Smoking
Dyslipidemia
Arterial hypertension
Diabetes mellitus
Family history of CAD
Personal history of CAD
Male sex
**Liver-Cirrhosis-Specific Risk Factors**
NAFLD etiology
Decompensated liver cirrhosis and hypercoagulable state
